# Seeing the unseen: hyperspectral imaging opens a new window into the root economics space

**DOI:** 10.1111/nph.71527

**Published:** 2026-08-20

**Authors:** Boris Rewald

**Affiliations:** ^1^ Department of Forest Protection and Wildlife Management, Faculty of Forestry and Wood Technology Mendel University in Brno Brno 613 00 Czech Republic

**Keywords:** belowground ecology, hyperspectral imaging, root economics space, root system heterogeneity, spectral trait prediction, woody fine roots

## Abstract

This article is a Commentary on Makita *et al*. (2026), **252**: 1816–1831.

Fine roots – the ‘hidden half’ of plants – regulate water, nutrient uptake and transport, underpin plant resource‐use strategies, and influence community and ecosystem processes (Freschet *et al*., 2021). Yet despite their ecological importance, roots remain considerably less studied than leaves and shoots, owing in part to methodological challenges. While advances in imaging technologies have transformed aboveground plant sciences over the past decade, from crop breeding and stress detection to forest monitoring (Fahlgren *et al*., 2015; Bian *et al*., 2022), root research still relies largely on labour‐intensive and time‐consuming manual measurements. In an article published in this issue of *New Phytologist*, Makita *et al*. (2026; pp. 1816–1831) demonstrate how hyperspectral imaging (HSI; Box 1) can be applied to living fine roots to characterize chemical, anatomical, and morphological traits, opening new opportunities for high‐throughput and spatially resolved root research.

Box 1 **What is hyperspectral imaging?**

Hyperspectral imaging (HSI) combines imaging and spectroscopy to collect both spatial and spectral information. Unlike spectroscopy, which measures a single averaged spectrum from a sample, HSI records a wavelength‐dependent reflectance pattern for every pixel in an image – its *spectral signature*. This signature arises from wavelength‐specific absorption and scattering of light within the sample and is influenced not only by its chemical composition and structure but also by its imaging platform configuration. Depending on the spectral range of the instrument, HSI can encompass the visible (VIS; *c*. 400–700 nm), near‐infrared (NIR; *c*. 700–1300 nm) and short‐wave infrared (SWIR; *c*. 1300–2500 nm) regions. The resulting dataset forms a three‐dimensional *datacube*, consisting of two spatial dimensions (x,y) and one spectral dimension (wavelength, λ). This pixel‐by‐pixel approach minimizes the loss of spatial information and enables the visualization of heterogeneity within samples (Morais *et al*., 2019).

*…Makita* et al. *show that comparable insights into within‐root‐system functional heterogeneity may be obtainable from comparatively simple HSI workflows applied directly to* in‐situ *sampled and rinsed woody roots*.

The study arrives at a pivotal moment in root ecology. Over the past decade, trait‐based frameworks have evolved from the one‐dimensional root economics spectrum towards the multidimensional root economics space (RES), in which root strategies are structured along at least two independent axes (Bergmann *et al*., 2020). One axis reflects a conservation trade‐off (fast vs slow resource use, e.g. high nitrogen (N) vs high tissue density (RTD)), while the collaboration axis reflects reliance on mycorrhizal partners (thick, cortex‐rich roots vs thin ‘do‐it‐yourself’ roots with high specific root length (SRL)). At the same time, root‐order‐based analyses have increasingly highlighted the substantial functional differentiation within root systems themselves. Distal, lower‐order roots such as tips primarily acquire resources and host mycorrhizal symbionts, whereas higher‐order roots increasingly contribute to transport, storage, and structural support. This differentiation can be viewed as an absorptive‐to‐transport continuum, in which root segments progressively shift from acquisition and symbiotic interaction towards transport and other functions with increasing root order. Different segments of a root system may therefore occupy distinct positions within the RES (McCormack *et al*., 2015; Tawa *et al*., 2026). Together, these conceptual advances highlight the need for approaches capable of characterizing functional variation across multiple biological scales.

Characterizing root strategies across species, environments and branching orders requires the measurement of multiple traits, a process that remains labour‐intensive and difficult to scale even when restricted to core traits such as SRL, root diameter (D), root tissue density, and N concentration (Matthus *et al*., 2025). Unlike leaf trait research, which continues to be transformed by spectroscopy‐based trait estimation (Burnett *et al*., 2021), root ecology has largely lacked comparable high‐throughput approaches. Consequently, the field's central challenge appears increasingly observational rather than conceptual. While recent studies have demonstrated the utility of HSI for (crop) root segmentation and classification tasks (Faehn *et al*., 2024; Baykalov *et al*., 2025; Lombardi *et al*., 2025), Makita *et al*. demonstrate that HSI can also be used for functional root trait characterization. Using fresh, intact fine‐root branches from 20 temperate tree species spanning contrasting phylogenetic groups and mycorrhizal associations, the authors evaluated whether spectral reflectance across visible–near‐infrared (VIS–NIR) and short‐wave infrared (SWIR) regions (Box 1) could predict a suite of morphological, anatomical, and chemical root traits closely related to the RES. Root diameter showed the highest predictive accuracy, while SRL, stele ratio, and several chemical traits were also estimated with useful precision across three root diameter classes, which broadly capture functional transitions associated with root branching order. Models on calibration data including SWIR wavebands often outperformed those relying solely on VIS–NIR wavebands, highlighting the importance of longer wavelengths for capturing variation in root structural and chemical properties, consistent with previous findings (Baykalov *et al*., 2025). Notably, validation models often benefited from the full spectral range, suggesting that complementary information was distributed across multiple regions of the VIS–NIR–SWIR spectrum. Indeed, the NIR region appeared to carry the strongest signal for species differentiation, particularly in roots > 0.5 mm diameter. If NIR reflectance is primarily governed by tissue structure, as often suggested in plant spectroscopy, this pattern may reflect interspecific differences in boundary‐tissue organization, including variation in the transition from rhizodermis‐ to periderm‐dominated root segments.

Importantly, the predicted root traits successfully reproduced the ordination of multidimensional trait space across diameter classes and phylogenetic × symbiont groups. In other words, spectral trait estimates recovered a remarkably similar RES to that obtained from directly measured traits, suggesting that high‐resolution hyperspectral imaging combined with partial least squares regression modelling (Burnett *et al*., 2021) has considerable potential to transform the measurement of key root traits (Matthus *et al*., 2025) from a labour‐intensive process into a scalable, high‐throughput methodology (Fig. 1). A key advance is therefore not merely the prediction of individual traits, but the recovery of the patterns of trait covariation that underpin root ecological strategies. That spectra from fresh woody fine roots contain sufficient information to reconstruct these multidimensional relationships is a central finding of the study. By showing that a single hyperspectral scan (after calibration) can replace multiple separate measurements, Makita *et al*. provide a promising pathway for scaling belowground trait ecology.

**Fig. 1:**
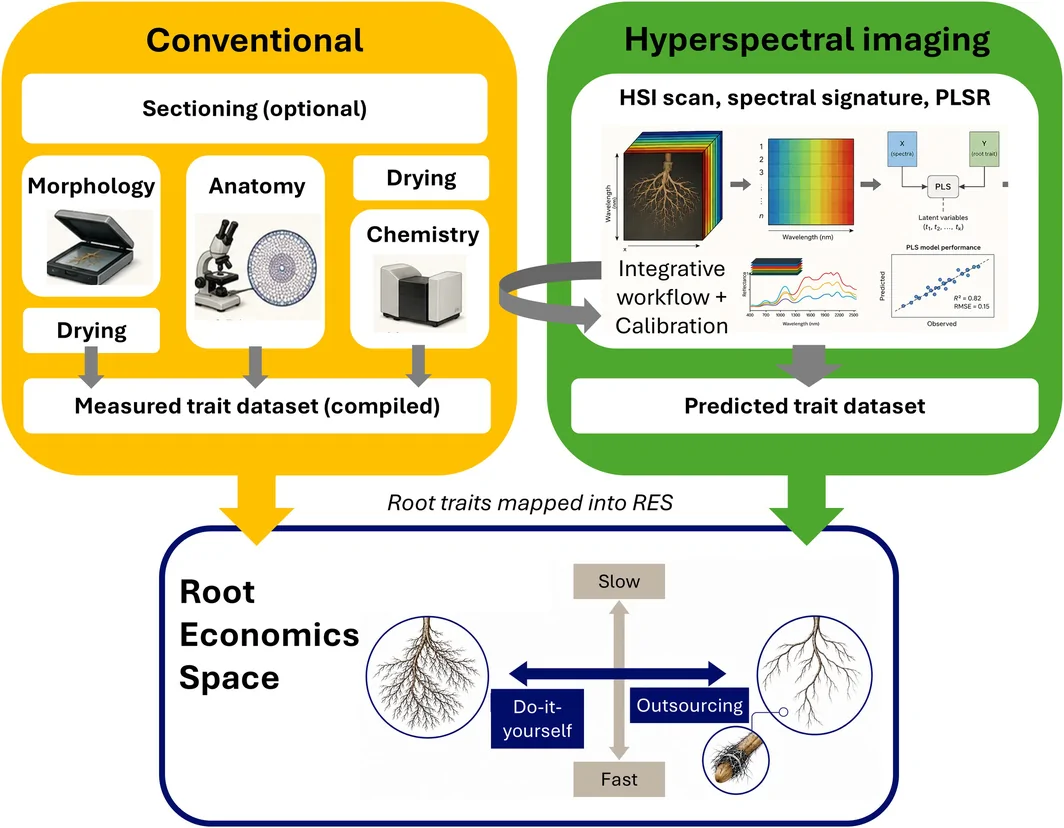
From conventional root trait measurements to hyperspectral imaging‐based (HSI) trait prediction. The conventional workflow (left) involves optional root sectioning into diameter classes or root orders, followed by separate morphological, anatomical, and chemical analyses, often requiring sample drying and multiple processing steps before compiling a root trait dataset. In contrast, the HSI workflow (right) integrates spectral data acquisition and analysis by generating a spectral data cube from a single scan, extracting spectral signatures, and using predictive models (e.g. partial least squares regression (PLSR)) to predict root traits against measured root traits. Although HSI reduces the need for extensive individual measurements, calibration with conventionally measured reference traits remains essential for reliable trait prediction. Both workflows ultimately generate data that can be used to position roots within the root economics space (RES), but HSI may provide a streamlined and (partially) automatable pathway that reduces the need for extensive individual measurements while capturing key dimensions of the RES.

Beyond mean trait prediction, Makita *et al*. also illustrate the potential for spatially explicit root ecology. Their final figure presents maps of predicted N concentrations along entire root branches, showing elevated N concentrations in distal absorptive root orders and declining concentrations towards higher‐order transport roots (McCormack *et al*., 2015). More importantly, these maps illustrate that positions within the RES vary continuously along root systems rather than being ‘mean’ species traits or confined to discrete categories such as diameter classes or root orders. While spatially explicit root phenotyping has advanced considerably through rhizotron‐based HSI platforms, particularly in crops (Rellán‐Álvarez *et al*., 2015; Faehn *et al*., 2024), Makita *et al*. show that comparable insights into within‐root‐system functional heterogeneity may be obtainable from comparatively simple HSI workflows applied directly to *in‐situ* sampled and rinsed woody roots.

Future studies may characterize HSI spectra of root segments with known positions along the absorptive‐to‐transport continuum, thereby linking spectral variation to underlying functional and developmental variation (Fig. 2). Ectomycorrhizal roots may represent a particularly interesting case for HSI because the fungal mantle forms an external layer around lower‐order roots and therefore directly contributes to the spectral signal. Combined with advances in deep‐learning‐based image analysis (Wang *et al*., 2026), HSI could enable a new generation of root‐segment‐specific ecology in which resource‐acquisition strategies are studied at the spatial scales at which they originate. Rather than treating fine‐root branches as functionally uniform, researchers may soon be able to map trait distributions within root systems and directly link particular functions to specific root segments. More fundamentally, such approaches may transform how we conceptualize the RES itself – from a framework based on mean species trait values to one that captures the spatially and temporally dynamic distribution, abundance, and functional properties of individual root segments within a root system. Importantly, the current practice of aggregating fine‐root traits can obscure the distinction between lower‐order absorptive roots and the trait values of the whole fine‐root branch, potentially masking substantial functional heterogeneity among root segments. Fig. 2 illustrates one possible future extension of this approach, in which individual root segments are positioned within the RES and integrated to derive dynamic branch‐level trait strategies. HSI therefore offers not only a means of accelerating trait measurements, but also a pathway towards spatially explicit and scalable representations of belowground functional diversity.

**Fig. 2:**
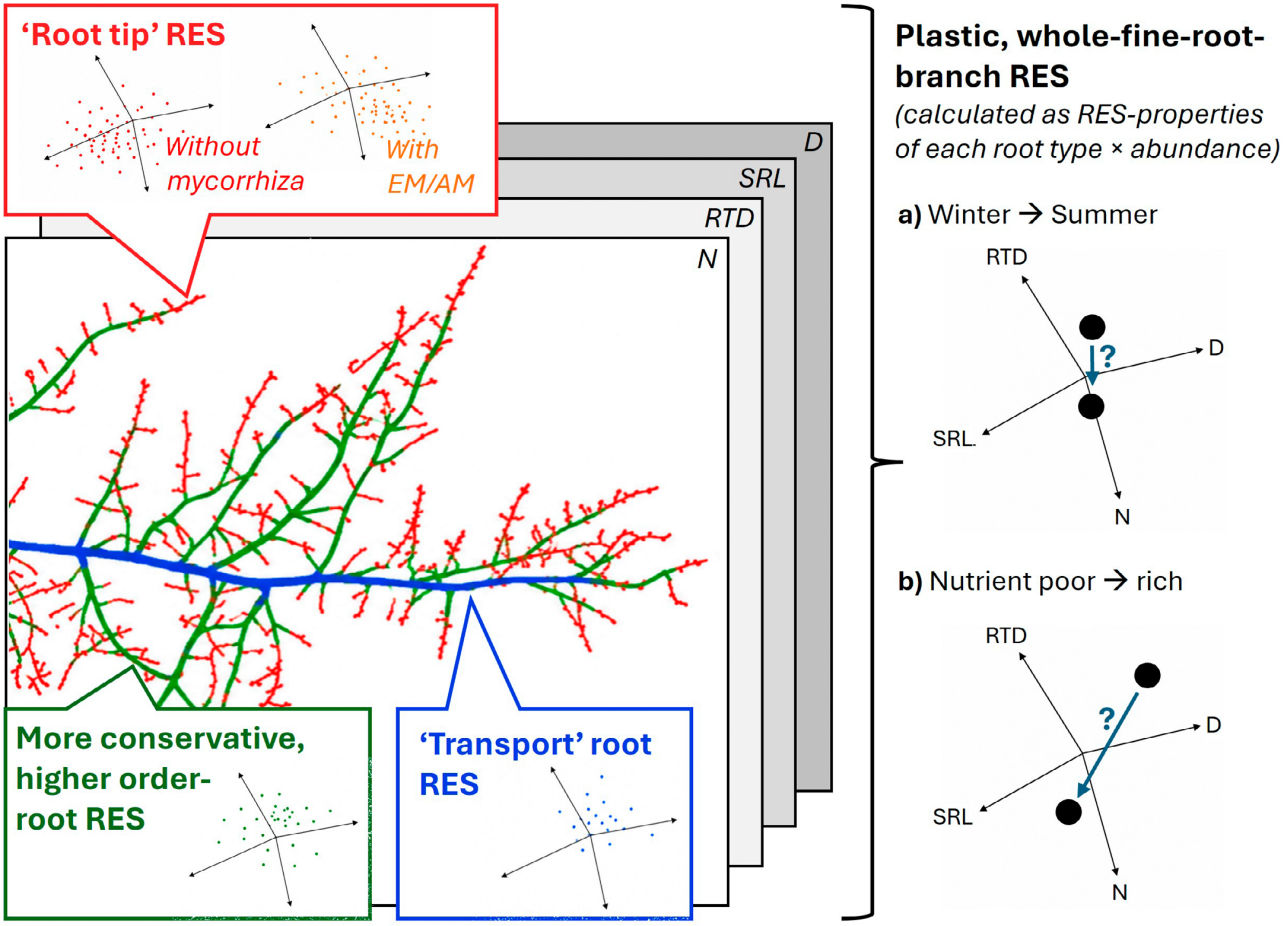
Hyperspectral imaging as a potential framework for mapping intact root systems into the root economics space (RES). Hyperspectral information may help to predict key root traits (N, root nitrogen content; RTD, root tissue density; SRL, specific root length; D, root diameter) and position individual root segments along the conservation and collaboration dimensions of the RES (left). Absorptive root tips and some lower‐order roots occupy the fast, ‘do‐it‐yourself’ region (red), mycorrhizal roots tend towards the collaborative region (orange), higher‐order fine roots occupy more conservative positions (green), and transport roots hold the highest RTD and D (blue). The RES position of an entire root branch (right) reflects the integrated trait values and relative abundance of its constituent segments. Because root branching and mycorrhizal colonization vary, for example, seasonally (a) and in response to resource availability (b), branch‐level RES positions may shift. By resolving trait variation among individual root segments, hyperspectral imaging may provide a mechanistic framework linking root‐system organization and plasticity to species‐level RES positions.

As with any emerging methodology, important challenges remain before HSI becomes a routine tool in root ecology. An important caveat is the potential collinearity between root diameter and other root traits, raising the possibility that some spectral signatures attributed to specific traits may instead reflect their covariance with diameter and associated differences in tissue structure. Furthermore, Makita *et al*. rightly point out that the high, and potentially variable, water content of fresh roots can mask absorption features and reduce signal quality. This challenge likely explains why early applications of root spectroscopy frequently relied on dried root material (Rewald & Meinen, 2013). Similarly, HSI primarily captures optical properties of root surface‐ and near‐surface tissues, with limited penetration into deeper anatomical structures. This may introduce uncertainty because spectral information originates primarily from outer tissues whereas laboratory analyses integrate the chemistry of the entire root cross‐section. Such scale mismatches may be particularly important for traits that vary strongly among tissues, such as lignin concentration. Furthermore, the influence of residual soil on root spectral signatures has yet to be systematically evaluated but could potentially explain the higher prediction error in fine‐root N than in leaves, as discussed by Makita *et al*. Future work should therefore focus on developing standardized sampling, storage, and spectral imaging and data‐processing protocols that account for these challenges while retaining the advantages of measuring living roots. A second challenge concerns biological generality and model transferability. Developing spectral libraries that encompass diverse species across environments will be essential if HSI is to achieve wider applicability. Whether models calibrated on the roots of one species pool, ecosystem, or imaging platform can be transferred across studies remains largely unknown. However, advances in leaf HSI provide valuable guidance for addressing these challenges (Burnett *et al*., 2021).

Yet these limitations should be viewed less as obstacles and more as opportunities. The long‐term promise of HSI is thus not only faster trait measurement, but also a new methodological framework capable of linking function and spatial organization within branched root systems. Just as hyperspectral imaging transformed aboveground plant ecology from laborious trait measurements to ecosystem‐scale trait mapping, HSI may now provide a comparable pathway for root research. In this sense, Makita *et al*.'s work represents an important step toward unravelling the ‘hidden half’, opening a new observational window into the spatial organization of belowground functional variation.

## Disclaimer

The New Phytologist Foundation remains neutral with regard to jurisdictional claims in maps and in any institutional affiliations.
